# Effects of High Starch and Supplementation of an Olive Extract on the Growth Performance, Hepatic Antioxidant Capacity and Lipid Metabolism of Largemouth Bass (*Micropterus salmoides*)

**DOI:** 10.3390/antiox11030577

**Published:** 2022-03-17

**Authors:** Xiaofang Liang, Pei Chen, Xiaoliang Wu, Shujuan Xing, Sofia Morais, Maolong He, Xu Gu, Min Xue

**Affiliations:** 1 National Aquafeed Safety Assessment Center, Institute of Feed Research, Chinese Academy of Agricultural Sciences, Beijing 100081, China; liangxiaofang01@caas.cn (X.L.); chen_pei1@ctg.com.cn (P.C.); 82101201182@caas.cn (X.W.); shujuan.xing@wur.nl (S.X.); guxu@caas.cn (X.G.); 2 Innovation Division, Animal Science Unit, Lucta S.A., 08193 Bellaterra, Spain; 3 Innovation Division, Lucta (Guangzhou) Flavours Co., Ltd., Guangzhou 510530, China; maolong.he@lucta.com

**Keywords:** largemouth bass, high starch, antioxidant, lipid metabolism, metabolic liver disease

## Abstract

An 8-week feeding trial was conducted to investigate the effects of high-starch diets and the supplementation of an olive extract (OE) on the growth performance, liver health and lipid metabolism of largemouth bass (*Micropterus salmoides*). Four isonitrogenous and isolipidic diets were prepared: two basal diets containing low (9.0%) and high (14.4%) levels of starch (named as LS and HS), and 0.125% OE was supplemented to each basal diet (named LSOE and HSOE). The results show that high-starch diets had significant negative effects on growth performance, with lower FR, SGR and higher FCR, whereas OE significantly lowered FCR, determined by two-way ANOVA analysis. High-starch diets induced oxidative stress, inflammatory response and liver function injury, with significant increases in the content of plasmatic AKP, AST, ALT, hepatic SOD and MDA, and up-regulation of hepatic *TNFα*, *IL1β*, and *TGFβ1* gene expression. In addition, a high-starch diet decreased the phosphorylation of AMPK and upregulated the expression of SREBP, together with higher hepatic liver lipid and HSI. The oxidative stress and lipid metabolism disorders indicate metabolic liver disease (MLD) of largemouth bass fed high-starch diets. Feeding on OE-supplemented diets increased the hepatic antioxidant capacity by decreasing the content of MDA and SOD. Fish fed the HSOE diet had an activated phosphorylation of JNK and decreased expression of pro-inflammatory *IL1β* compared with those fed the HS diet, which strongly indicated that the degree of inflammatory responses was reduced after OE supplementation. Interestingly, this study demonstrated that OE regulates hepatic lipid metabolism in fish by inhibiting the expression of hepatic lipogenesis genes (*ACC1* and *FASN*) and promoting lipolysis (*ATGL*) and β-oxidation (*CPT1α*) to prevent TG accumulation. In conclusion, high-starch feed induced oxidative stress and lipid metabolic disorder of largemouth bass, while supplementation with OE improved its antioxidant capacity, anti-inflammatory responses and lipid metabolism. However, hepatic histopathological results suggested that OE supplementation could not completely repair the MLD caused by the high level of starch in largemouth bass.

## 1. Introduction

World aquaculture production has increased tremendously over the past years. However, due to intensive farming practices, metabolic diseases of fish have posed a great threat to fish production, causing heavy economic losses. Largemouth bass (*Micropterus salmoides*) is a North America-native carnivorous species, which has been one of the most widely cultured and consumed fish species in China because of its rapid growth, good flesh quality, and high market value. Its production in 2020 was up to 600 thousand tons in China. As a typical carnivorous fish, *M. salmoides* was traditionally fed on chilled fish; however, with the requirements for sustainable development of aquaculture, more and more formulated diets are used nowadays. Starch is necessary as a binder and sweller during extrusion processing of formulated, pelleted feeds [1]. However, excess dietary starch in carnivorous fish diets can induce hyperglycemia, glycogen and lipid accumulation, and chronic inflammation response, as well as compromising the immune function and antioxidant capabilities, eventually resulting in metabolic liver disease (MLD) [2,3,4]. 

To develop environmentally friendly aquaculture and ensure food safety, various non-antibiotic feed additives have been used to improve animal health [5]. Different plant extracts have been reported to act as immunostimulants and to have antibacterial and anti-parasitic (virus, protozoans, monogeneans) properties in aquaculture due to the presence of active molecules such as alkaloids, terpenoids, saponins and flavonoids [6]. Recently, olive extracts (OEs), either the byproduct of olive oil production or leaf extract (OLE), have been proven as natural products playing very important roles as antimicrobial, anti-inflammatory, antiviral and anti-tumor compounds, in which particular emphasis is placed on the antioxidant activity of the extracts [7]. At present, research on the application of olive extracts in aquaculture is mainly focused on OLE. The main active components of OLE are polyphenols, in which the most abundant is oleuropein [8]. Dietary OLE alters some immune gene expression levels and disease resistance to *Yersinia ruckeri* infection in rainbow trout (*Oncorhynchus mykiss*) [9]. Furthermore, dietary supplementation of OLE can enhance the growth performance of common carp (*Cyprinus carpio*) by activating digestive enzyme activity in the intestine and increasing the expression level of several genes (*GH* and *IGF-I*) related to growth in the brain, liver, head kidney and muscle [10]. On the other hand, the active components of OE derived (as byproduct) from olive oil production also include triterpenes, such as maslinic acid and oleanolic acid, which are bioactive components with anti-oxidative, anti-inflammatory and hepatoprotective activities [11]. Triterpenes are involved in the regulation of oxidative status through the reduction in ROS, raising SOD and CAT activities, the suppression of nuclear factor-κB (NF-κB), and the prevention of lipid peroxidation [7,12,13,14]. OE has been demonstrated as an immunopotentiator in Black seabream (*Acanthopagrus schlegelii*) [7]. In addition, research performed in mammals has established that triterpene-enriched OE is involved in lipid regulation. For example, OE treatment has been proved to decrease hepatic lipid accumulation by regulating lipid metabolism in vivo and in vitro in mice [15]. Therefore, we hypothesized that OE has the potential to prevent or reduce MLD caused by a high-starch diet in aquatic animals. To our knowledge, information regarding the roles of OE in MLD in largemouth bass have not been reported yet.

The objective of the present study was to investigate effects of high-starch diets and the supplementation of OE on the growth performance, hepatic antioxidant capacity and lipid metabolism of largemouth bass. The results of this study provide insights into the environmentally friendly prevention of MLD in carnivorous fish.

## 2. Materials and Methods

### 2.1. Growth Trial and Sample Collection

Two isonitrogenous and isoenergetic experimental diets with 9.0% (low starch, named as LS) and 14.4% (high starch, named as HS) starch were prepared, and 0.125% OE (Lucta S.A., Barcelona, Spain) was added to these two basal diets, respectively (named as LSOE and HSOE). The OE product was prepared from the olive cake remaining from typical two-phase olive oil production, with a standardized composition (determined by HPLC) of minimum 7.5% triterpenes (maslinic acid and oleanolic acid). The product also contains > 2% protein, > 5% fat and > 30% fiber. The feed ingredients were ground into fine powder through a 247 μm mesh. Each diet was processed into 3 mm-diameter floating pellets under the following extrusion condition: feeding section (90 °C/5 s), compression section (130 °C/5 s), and metering section (150 °C/4 s), using a Twin-screwed extruder (EXT50A, YangGong Machine, Beijing, China) according to our previous studies [4,16]. All diets were air-dried at room temperature and stored at −20 °C until use. The diet formulations and analyzed chemical compositions are shown in Table 1.

Largemouth bass were obtained from a commercial aquafarm (Tangshan, Hebei, China). All fish were acclimated and fed the control experimental diet (LS diet) for 4 weeks before the formal feeding trial. After a 24 h fasting period, fish (initial body weight = 35.98 ± 0.21 g) were distributed into 12 cylindrical plastic tanks (capacity: 256 L) with three replicates per treatment and 20 fish per tank, and each diet was randomly assigned to 12 tanks. Fish were fed to apparent satiation twice daily at 08:00 h and 17:00 h. During the experiment, water temperature was maintained at 21–25 °C, pH at 7.2–8.0, dissolved oxygen > 6.0 mg/L, and ammonia-N < 0.3 mg/L.

Growth performance factors (final body weight (FBW), survival rate (SR), specific growth rate (SGR), feed conversion ratio (FCR), feeding rate (FR)) were determined by batch weighing the fish at the end of the 8 weeks after starvation for 24 h. All the sampled fish were anesthetized with chlorobutanol (300 mg/mL). Individual body weight, body length, viscera, liver and visceral adipose tissue weight of four fish in each tank were recorded to calculate the condition factor (CF), viscerosomatic index (VSI), hepatosomatic index (HSI) and visceral adipose index (VAI). Blood was rapidly drawn from the caudal vein and centrifuged (4000× *g*, 10 min, 4 °C) to obtain plasma for the analysis of hematological parameters. Four liver samples from each tank were dissected and immediately frozen in liquid nitrogen, and kept at −80 °C for mRNA isolation and tissue homogenate analysis until used. Four liver samples near the bile duct in each tank were fixed in 4% paraformaldehyde (Solarbio, China) for histology determination. The rest of the livers in each tank were pooled into zip-lock bags and then stored at −20 °C for the assay of crude lipids.

### 2.2. Chemical Composition Analysis of Diets

The crude protein, crude lipid, crude ash, moisture, starch, and gross energy contents of experimental diets and whole body of fish were analyzed according to standard methods as previously described [4,16].

### 2.3. Plasma and Hepatic Homogenate Parameters 

The content of plasma glucose, triglycerides (TG), total cholesterol (TC), high-density lipoprotein cholesterol (HDL-C), low-density lipoprotein cholesterol (LDL-C), total bile acids (TBA), alkaline phosphatase (AKP), aspartate aminotransferase (AST), alanine aminotransferase (ALT), and hepatic TG, TC, TBA, HDL-C, LDL-C, malondialdehyde (MDA) and activities of total antioxidant capacity (T-AOC) and glutathione peroxidase (GSH-Px), superoxide dismutase (SOD), and catalase (CAT) were determined by commercial assay kits (Nanjing Jiancheng Co., Nanjing, China) following the manufacturer’s protocols. Reactive oxygen species (ROS) was determined using a commercial kit (Jiangsu Meimian industrial Co., Ltd., Yancheng, China). 

### 2.4. Hepatic Histopathological and Immunofluorescence Examination

Liver samples were fixed, dehydrated, embedded, and stained for hematoxylin and eosin (H&E) as previously described [4,16]. The results of HE staining were observed using light microscopy (DM2500, Leica, Weztlar, Germany). Immunohistochemistry for NF-κB was conducted as previously described [16]. Briefly, sections were incubated with polyclonal NF-κB (#8242, CST, Boston, USA) overnight at 4 °C. The results were observed using a high-resolution living cell imaging system (DeltaVision, GE, Boston, USA).

### 2.5. Quantitative Real-Time PCR 

Total RNA extraction and cDNA synthesis were carried out as described previously [4,16]. The qPCR analysis was performed using a CFX96TM Real-Time System (Bio-Rad, CA, USA) using iTaqTM Universal SYBR^®^ Green Supermix (Bio-Rad, CA, USA). Each sample was run in triplicate and analyzed using the 2^−ΔΔCt^ method. *EF1α* was used as an endogenous reference gene. Primer sequences are shown in Table 2.

### 2.6. Western Blot 

Protein extraction of the liver samples was carried out as described previously [4]. Protein concentration was measured using a BCA Protein Quantification Kit (Bio-Rad, CA, USA). The primary antibodies used were β-tubulin (#2146, CST, Boston, USA), ERK1/2 (#4695, CST, Boston, USA), P-ERK1/2(Thr202/Tyr204) (#4370, CST, Boston, USA), AMPKα (#A11184, Abclonal, Wuhan, China), P-AMPKα (AMPKα1-Thr-183/AMPKα2-Thr172) (#AP0432, Abclonal, Wuhan, China), JNK (#9252, CST, Boston, USA), and P-JNK (Thr-183/Tyr185) (#9252, Genetex, CA, USA). Automated Western blots were performed on a JessTM system (Protein Simple) using pre-filled plates (12–230 kDa) according to the manufacturer’s standard instructions.

### 2.7. Statistical Analysis

All data were presented as the mean value ± standard error of the mean (S.E.M). Statistical analyses were performed using the SPSS software version 22.0 for Windows (IBM Inc., New York, USA). All data means were analyzed after homogeneity of variances were tested. Two-way ANOVA was used to analyze the significant differences among treatment means based on starch levels (9.0 and 14.4%) and OE levels (0 and 0.125%). Meanwhile, one-way ANOVA was also performed for the data analysis of four groups in figures. *p* < 0.05 was considered significantly different. The graphics were drawn using GraphPad Prism 7.0 (GraphPad Software Inc., CA, USA).

## 3. Results

### 3.1. Growth Performance and Morphometric Parameters

The results of growth performance and morphometric parameters are presented in Table 3 and Table 4. The SR of largemouth bass was the same in all groups (*p* > 0.05). According to the two-way ANOVA analysis, high-starch diets significantly reduced the FBW, SGR, FR and FCR compared with low-starch diets (*p* < 0.05). There were no significant differences in FBW, SGR, and FR after adding 0.125% OE to the diets (*p* > 0.05). However, supplementation of OE significantly decreased the FCR (*p* < 0.05). Both a high starch level and OE supplementation significantly increased the HSI, while OE supplementation significantly decreased the CF and high starch significantly increased the VSI (*p* < 0.05). Both a high starch level and OE supplementation had no effect on the crude protein content of the whole body (*p* > 0.05). OE supplementation significantly increased the crude lipid level of the whole body (*p* < 0.05) (Table 5). 

### 3.2. Hematological and Liver Functions Parameters

The hematological and liver function parameters of largemouth bass are presented in Table 6 and Table 7. No significant differences were observed in plasma glucose, TG, TC, HDL-C, LDL-C between largemouth bass fed low-starch and high-starch diets (*p* > 0.05). OE supplementation significantly increased plasma TC, which was mainly induced by the increase in HDL-C (*p* < 0.05), and plasma LDL-C/TC was decreased with 0.125% OE supplementation (*p* < 0.05). Moreover, high levels of dietary starch led to abnormal liver function with significantly higher AST, ALT and AKP, and supplementation with OE significantly decreased ALT in plasma, which indicated that OE may improve liver function.

### 3.3. Hepatic Antioxidant Responses

The contents of hepatic ROS were significantly higher, and those of SOD and CAT were lower in the group fed the high-starch diet than those fed the low-starch diet. The supplementation with OE significantly decreased the contents of hepatic MDA, SOD and CAT, and SOD/MDA was significantly increased (*p* < 0.05), which indicated that OE may improve hepatic antioxidant capacity (Table 8).

### 3.4. Hepatic Pathological Examination—Histology

**Figure 1:**
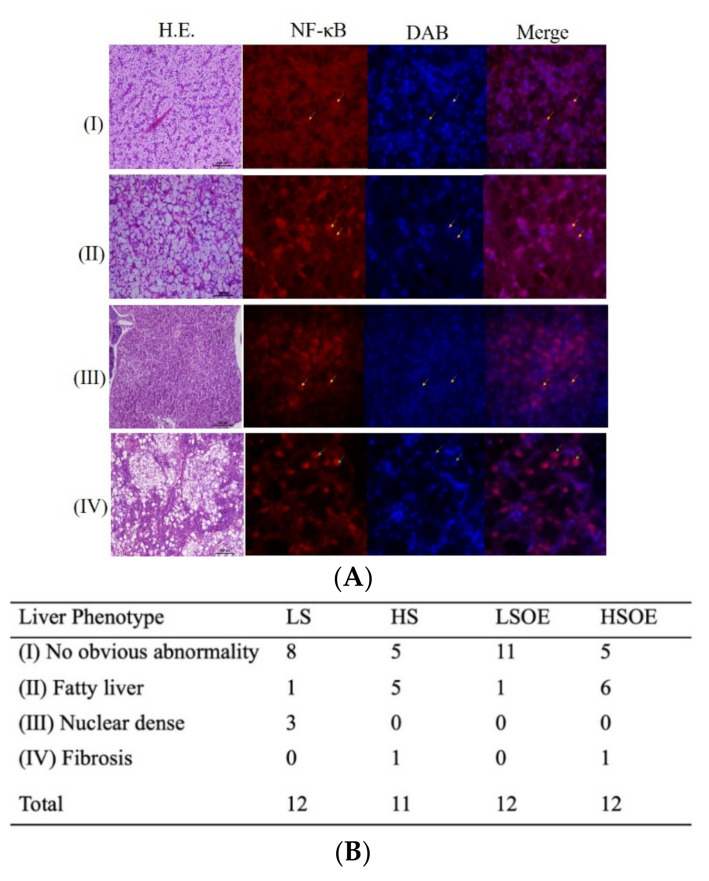
Effects of different diets on hepatic histopathological and inflammatory responses of largemouth bass. (**A**) Four phenotypes of hepatic histopathological examination with symptoms from light to heavy by HE staining for histology examination. Inflammatory response signals of NF-κB were lower (marked by yellow arrows) and mainly (marked by green arrows) expressed in the nucleus (marked with DAPI in blue color) (bar = 15 μm), in which (I) no obvious abnormity, (II) fatty liver, (Ⅲ) nuclear dense tissue, and (IV) hepatic fibrosis symptoms were observed. (**B**) Statistical results of liver phenotypes (n = 12). Since the samples were damaged during the embedding process, the number of slices was less than 12 of the HS group.

The hepatic histopathological examination results of each group are shown in Figure 1. Four phenotypes of hepatic histopathological examination were defined, with symptoms from light to severe, by H&E staining and immunofluorescence signaling for NF-κB (Figure 1A). Phenotype I showed normal hepatocytes with well-shaped cells and low expression of NF-κB in the nucleus. Phenotype II defined fatty liver tissues with enlarged and vacuolated cells and low expression of NF-κB in the nucleus. Phenotype Ⅲ defined nuclear dense tissues, which is usually a precursor to liver fibrosis, with unclear liver cord and low expression of NF-κB in the nucleus. Phenotype IV defined liver fibrosis, with severe vacuolation along with hepatic fibrosis symptoms and NF-κB mainly expressed in the nucleus. Twelve samples were observed in each group (except eleven samples in HS group). There were eight and eleven samples generally normal (phenotype I) in the LS and LSOE group. However, only five samples were generally normal in the HS and HSOE group. Fish in the HS and HSOE groups showed a high proportion of fatty liver (five to six samples) and even a fibrosis (one sample) phenotype (Figure 1B). 

### 3.5. Hepatic Proliferation and Inflammation Responses

**Figure 2:**
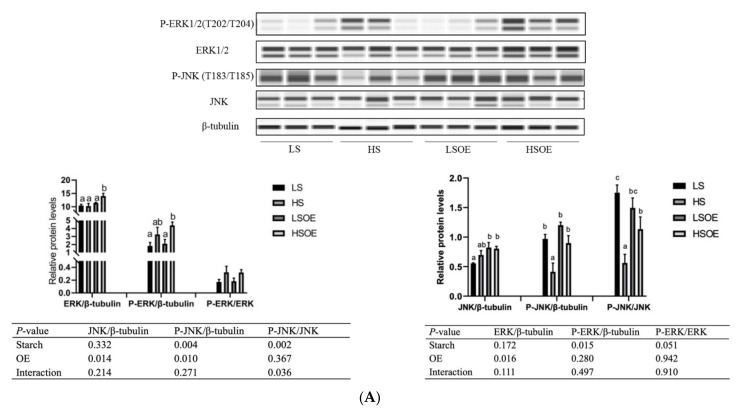

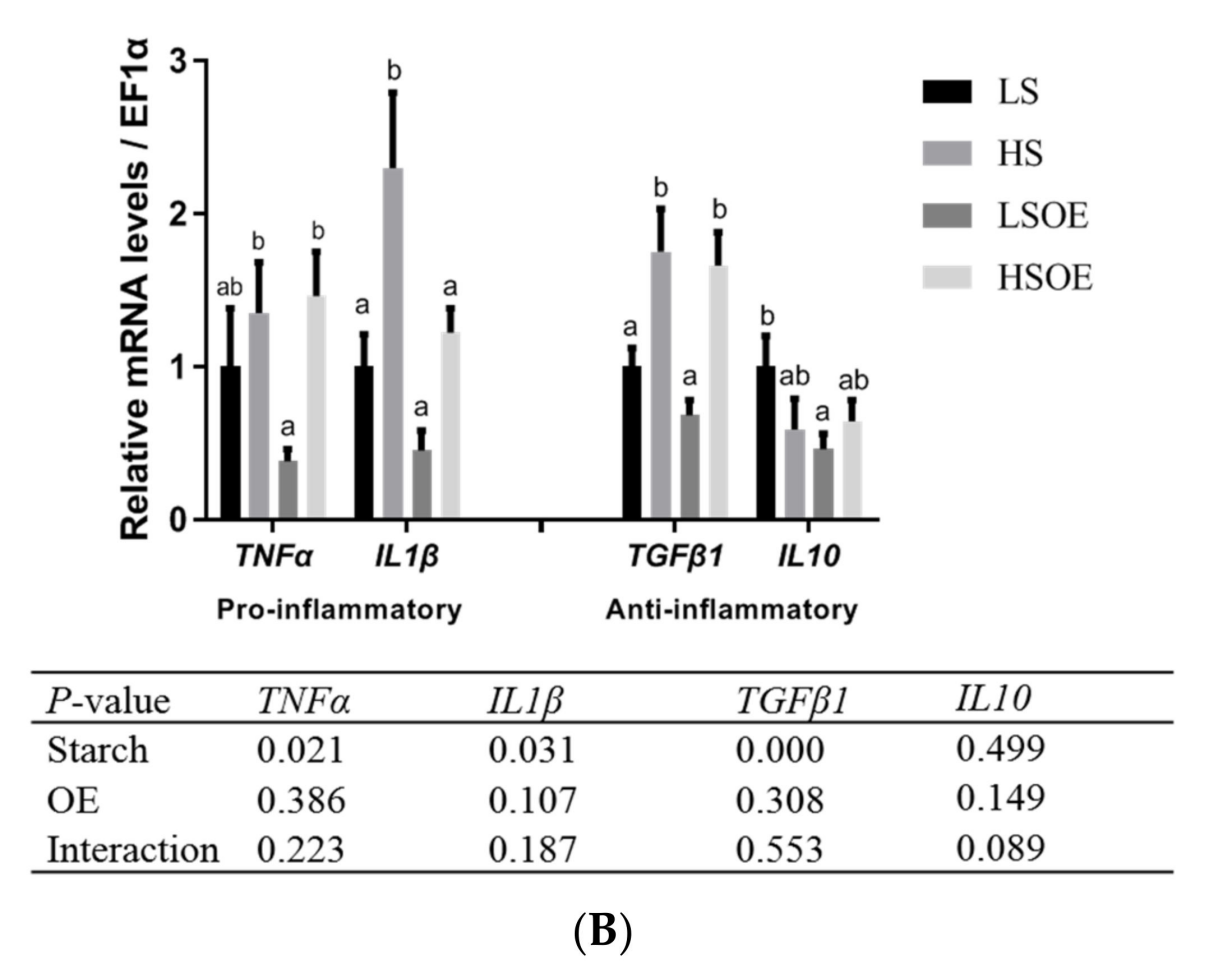
Effects of different diets on hepatic proliferation and inflammatory responses of largemouth bass, (**A**) Western blot of P-ERK, ERK, P-JNK and JNK in the liver (n = 3). (**B**) Effects of different diets on the transcriptional levels of hepatic pro- and anti-inflammation-related genes (n = 8). Both one-way ANOVA and two-way ANOVA statistics were analyzed. Differences were regarded as significant when *p* < 0.05 (n = 8). Values marked with “a, b and c” are significantly different according to one-way ANOVA.

As shown in Figure 2A, the phosphorylated ERK (P-ERK(Thr202/Tyr204)) levels in liver tissues were significantly higher in the high-starch groups than in the low-starch groups (*p* < 0.05). Simultaneously, the P-ERK/ERK ratio showed a tendency towards an increase in the high-starch groups. Supplementation with OE significantly increased ERK levels (*p* < 0.05), but had no significant effect on P-ERK and P-ERK/ERK levels (*p* > 0.05). Phosphorylated JNK (P-JNK(Thr183/Tyr185)) levels, and the P-JNK/JNK ratio in liver tissues, were significantly lower in the high-starch groups than in the low-starch groups (*p* < 0.05). Adding OE significantly decreased phosphorylated JNK (P-JNK) levels (*p* < 0.05), but had no significant effect on P-JNK(Thr183/Tyr185)/JNK levels (*p* > 0.05). Moreover, One-way ANOVA analysis showed that the ratio of P-JNK/JNK was significantly up-regulated in the HSOE group compared to the HS group (*p* < 0.05). We also observed a significant up-regulation of mRNA levels of pro-inflammatory cytokines (TNFα, IL1β) and anti-inflammatory cytokines (TGFβ1) in the high starch groups (*p* < 0.05), while supplementation with OE had no significant effect on the expression of inflammatory cytokines by two-way ANOVA (*p* > 0.05). However, one-way ANOVA analysis showed that the mRNA level of IL1β was significantly decreased in the HSOE group compared with the HS group (*p* < 0.05) (Figure 2B). The results show that high levels of dietary starch induced inflammatory response in largemouth bass, and that adding OE in the high-starch diets could reduce the expression of pro-inflammatory factors to a certain extent, which was achieved by activating the phosphorylation of JNK.

### 3.6. Hepatic Lipid Metabolism

As shown in Table 9, no significant differences were observed in hepatic TG, TC, LDL-C and TBA between largemouth bass fed low-starch and high-starch diets (*p* > 0.05). High-starch diets induced a higher content of lipid in the liver (*p* < 0.05). Supplementation of OE significantly decreased hepatic TG, LDL-C, LDL-C/TC and TBA (*p* < 0.05). Although the two-way ANOVA results showed that OE had no significant effect on the level of liver lipids (*p* > 0.05), there was a clear reduction in hepatic lipids after adding OE to the low starch diet.

**Figure 3:**
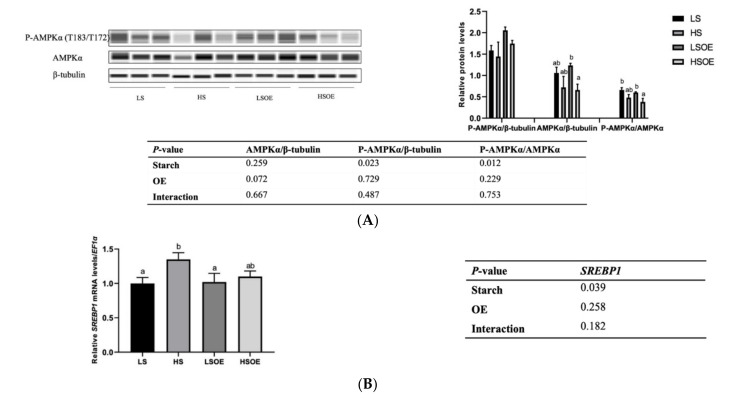

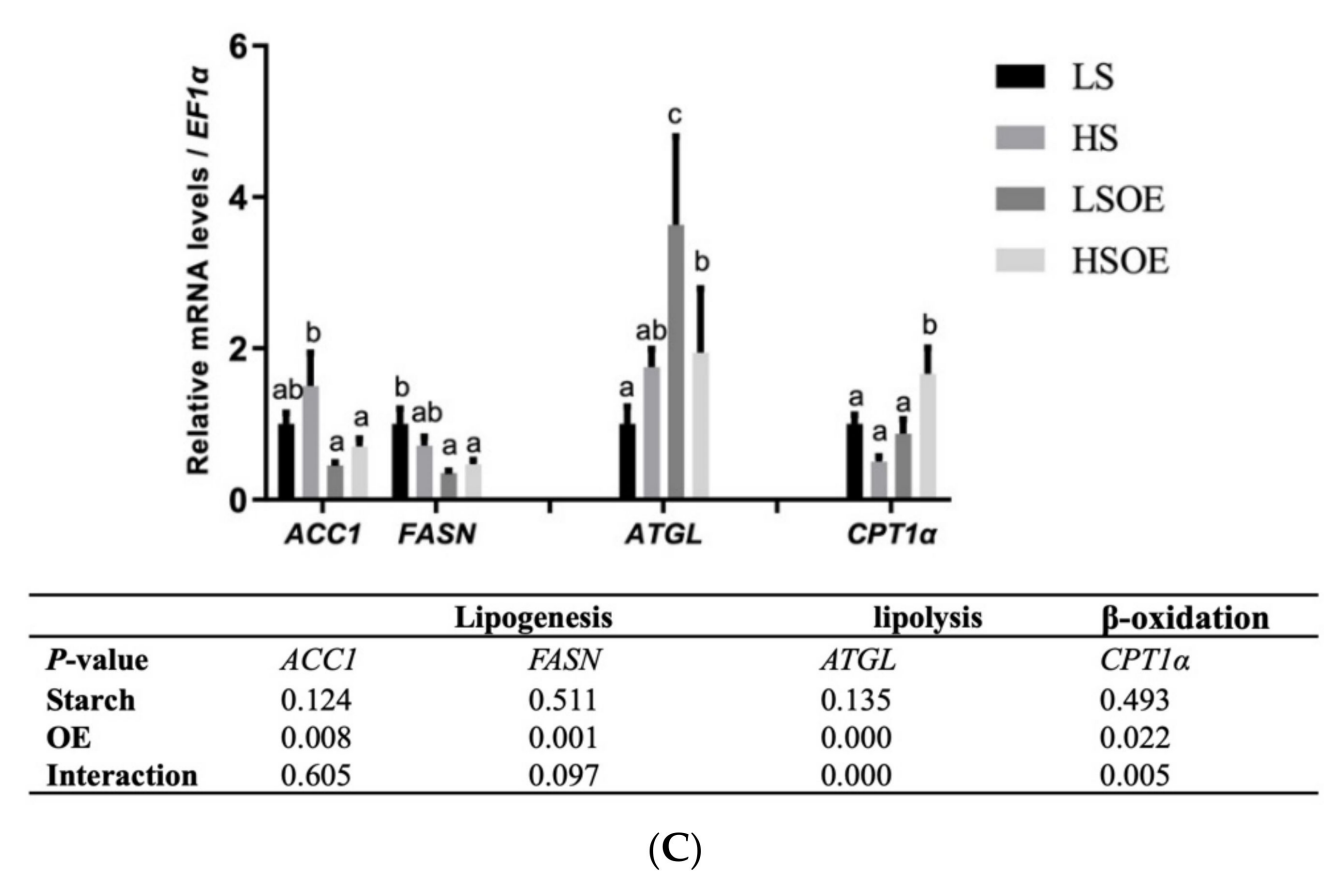
Effects of different diets on the hepatic lipid metabolism of largemouth bass. (**A**) Western blot of P-AMPK and AMPK in the liver (n = 3). (**B**) Transcriptional levels of *SREBP1* (n = 8). (**C**) Transcriptional levels of hepatic FA synthesis (*ACC1* and *FASN*), TG hydrolysis (*ATGL*), and β-oxidation (*CPT1α*) related genes (n = 8). Both one-way ANOVA and two-way ANOVA statistics were analyzed. Differences were regarded as significant when *p* < 0.05 (n = 8). Values marked with “a, b and c” are significantly different according to one-way ANOVA.

As shown in Figure 3A, a high starch level decreased the phosphorylated AMPKα (P-AMPKα (AMPKα1 T183/AMPKα2 T172)) and P-AMPKα/AMPKα levels (*p* < 0.05). In terms of mRNA levels, high-starch diets significantly increased *SREBP1* compared with diets containing low starch (*p* < 0.05) (Figure 3B). On the other hand, supplementation with OE had a great influence on lipid metabolism. In particular, mRNA levels of hepatic fatty acid synthesis-related genes (*ACC1* and *FASN*) were significantly down-regulated, and TG hydrolysis (*ATGL*) and fatty acid β-oxidation (*CPT1α*)-related genes were significantly up-regulated by OE supplementation (*p* < 0.05) (Figure 3C).

## 4. Discussion

Starch is typically used as a technical ingredient (stabilizer and swelling agent) in the manufacture of aquatic feeds, and is sometimes used as a cheap energy source in diets for some fish species. However, carnivorous fish having a metabolism adapted to diets low in carbohydrates exhibit symptoms of glucose intolerance after the intake of high-starch diets [17,18]. Previous studies showed that largemouth bass had poor starch utilization capacity, and high dietary inclusion of digestible carbohydrate (over 10%) was recognized as the primary factor inducing MLD [19,20]. Excess dietary starch induced hyperglycemia, glycogen and lipid accumulation, chronic inflammation, and reduced immune functions and antioxidant capabilities, causing MLD in largemouth bass [2,4]. The present study indicated that, compared with low-starch feed (9.0%), high-starch (14.4%) feed decreased the growth performance of largemouth bass and induced liver lipid accumulation, inflammatory response, oxidative stress, liver function injury and higher hepatosomatic index, which is similar to the nonalcoholic steatohepatitis symptom (NASH). Matsumoto et al. developed a NASH model in the ricefish medaka (*Oryzias latipes*), which is based on feeding the fish a high-fat diet (HFD). Medaka that are fed a HFD exhibited macrovesicular fat deposition and liver dysfunction [21]. In aquaculture, various additives are commonly added to the diets to improve nutrient utilization, growth performance and survival of cultured fish, such as probiotics, yeast, amino acids, antioxidants, enzymes, plant extracts and certain organic acids/salts and so on [22,23,24]. Among these, plants extracts have a broad utilization for growth promotion and appetite stimulation [25]. For instance, garlic and ginger increased SGR and WG, and decreased FCR in rainbow trout [26,27]. Similar effects have been observed in Nile tilapia (*Oreochromis niloticus*) fed diets including extracts of ginseng (Ginsana^®^ G115) [28] or limonene and thymol [29], and in juvenile olive flounder (*Paralichthys olivaceus*) fed diets including extract of green tea [30]. Growth performance and expression levels of growth-related genes in common carp (*Cyprinus carpio*) were enhanced after feeding diets with 0.10–0.25% OLE, but decreased after feeding diets with high levels of OLE (0.50–1.00%) [10]. Some studies also demonstrated that dietary supplementation of 0.00–1.00% of OLE did not affect growth performance and feed utilization in rainbow trout [9]. In this study, 0.125% OE had no significant effect on the weight gain of largemouth bass, but FCR was clearly reduced, leading to decreases in the farming feed cost of largemouth bass.

To avoid metabolic stress, such as that potentially caused by high carbohydrate, organisms rely on an antioxidant protection system to prevent oxidative injury. SOD enzymes control the levels of a variety of ROS and reactive nitrogen, thus limiting the potential toxicity of these molecules and controlling broad aspects of cellular life that are regulated by their signaling functions [31]. MDA is the product from the peroxidation of n-3 and n-6 polyunsaturated fatty acids, and has a strong biotoxicity to cells [32]. Our study suggested that 14.4% starch in the diet reduced the hepatic antioxidant capacity of largemouth bass, with higher ROS and lower CAT and SOD levels, which was consistent with previous studies showing that different starch sources and levels significantly affected the antioxidant status of largemouth bass [19,33]. Significant reductions in SOD, CAT and GSH-Px activities, and an increase in MDA content, were detected in the liver of largemouth bass when dietary corn starch level increased from 0 to 25% [19]. In this study, the increase in ROS with rising starch level, with no differences observed after supplementation with OE, may be the main reason for the decrease in SOD. OE inclusion strongly decreased the hepatic MDA content in both high- and low-starch diets. The lower MDA content may be attributed to the lower oxidative stress and inflammatory degree, which can be demonstrated by the decreased SOD activity observed in this study. Moreover, triterpenes possess anti-inflammatory activity and antioxidant protection properties in vivo [34] and in vitro [35]. In recent years, more and more studies have proved that triterpenes play apoptotic roles against tumor cells, but a principal feature of these compounds is their antioxidant effect [11]. Therefore, triterpene-rich olive extracts are considered a natural source of antioxidant compounds for the prevention of fish diseases related to cell oxidative damage.

At present, the most widely reported application of olive extracts in fish is related to their immunomodulatory effects. Dietary supplementation of 0.1% OLE increased the expression levels of immune-associated genes (*IL-1β* in head kidney tissue and *TNF-α* in spleen tissue) and enhanced the survival rate of common carp juveniles by inhibiting the pathogenicity of *E. tarda* [10]. Adding 0.1% OLE to a rainbow trout’s diet activated the expression of immune-related genes in the spleen tissue, including *TNFα*, *IL1-β* and *IL-8*, although the expression levels of these genes decreased with higher doses of OLE, such as 0.25, 0.5, and 1.0% [9]. Triterpene-enriched OE acted as an immunopotentiator in Black Sea bream (*Acanthopagrus schlegelii*), repairing the hampered immune response induced by cadmium exposure [7]. Moreover, OLE could be applied in the control and prevention of white spot virus syndrome in white leg shrimp (*Penaeus vannamei*) [36]. In this study, increased expression levels of *IL1β*, *TNFα* and *TGFβ1* suggested that high starch contents activated the immune response of largemouth bass, while supplementation with OE in the diet containing high starch level reduced transcript levels of pro-inflammatory gene *IL1β* and expression of anti-inflammatory genes remained at a high level.

Oxidative stress induced by oxidized fish oil activated NF-κB signaling and release of inflammatory cytokines in *Megalobrama amblycephala* and *Rhynchocypris lagowskii* Dybowski [37,38]. In mammals, triterpenes have been shown to promote an anti-inflammatory status by suppressing nuclear NF-κB activity [39,40]. In fact, NF-κB pathway is a double-edged sword in the inflammatory response. Once the activity of NF-κB is abnormally increased or continuously activated, it shows an anti-apoptotic effect by inducing anti-inflammatory response and promoting the expression of target genes related to cell proliferation [41]. MAPK family member ERK1/2 plays an important role in cellular proliferation and differentiation. Protein phosphorylation is a process of protein post-translational modification. Usually, the ERK1/2 is located in the cytoplasm and quickly passes through the nuclear membrane once phosphorylated, and then activates transcription factors to further regulate the transcription of their respective target genes, causing changes in the expression or activity of specific proteins and ultimately regulating cell biological function [42]. A high starch level induced liver fibrosis and increased NF-κB in the nucleus, and the phosphorylation level of ERK1/2 showed an increasing trend, which suggests that the fish fed high starch diet was under a “self-repair” status. In the study, OE increased the total ERK1/2 expression, which may be regulated by the post-transcriptional translation of growth factor receptors from upstream signals (or protein tyrosine kinase receptors); however, phosphorylation activation is mainly reflected in the starch level rather than the presence of OE supplementation. In addition, another MAPK family member the JNK signal transduction pathway has been implicated in cellular stress, inflammation and apoptosis. The demethylating substance betaine could repair alcoholic and non-alcoholic fatty liver disease (NAFLD) by inhibiting JNK-mediated signaling [43]. Indeed, JNK plays a dual role in the development of hepatocellular carcinoma. JNK promotes an inflammatory hepatic environment that supports tumor development, but JNK deficiency in hepatocytes increased the tumor burden, and so also functions in hepatocytes to reduce tumor development [44]. Our results show that the high starch diet inhibited JNK phosphorylation, which could protect hepatocytes from apoptosis. On the other hand, the addition of OE to the high-starch diet promoted the activation (phosphorylation) of the JNK pathway in largemouth bass, concomitantly with the decrease in expression level of the proinflammatory factor *IL1β*. However, hepatic histopathological analysis did not show significant effects of OE supplementation on the repair of liver injury induced by the high starch diet, which may be a result of the short feeding time (eight weeks) in the study.

Hepatic lipid content and HSI are important indicators of liver health in fish. In this study, we demonstrated that a high-starch diet led to an over-accumulation of lipid and enlarged liver in largemouth bass, with a higher content of hepatic lipid and HSI. In addition, our results suggested that both AMPK and SREBP were involved in the over-accumulation of lipid. Up-regulation of SREBP increases the uptake and absorption of glycerol by hepatocytes and enhances the synthesis of fatty acids and cholesterol [45]. When the contents of free fatty acids, TG and TC exceed the adaptative capacity of the body, further accumulation in hepatocytes then leads to an overproduction of ROS, cytokines and inflammatory factors, which is the most common pathogenesis pathway of NAFLD [46]. AMPK, a kinase directly targeting SREBP-1, inhibits SREBP-1 cleavage and intranuclear translocation, and suppresses the expression of SREBP-1 target genes in hepatocytes exposed to high levels of glucose, thus decreasing lipogenesis [47]. In the present study, the high-starch diet activated AMPK, as observed by a decrease in phosphorylation of AMPK, which further upregulated the expression of *SREBP-1*. OE showed a tendency to decrease *SREBP-1* expression in the HS diet.

In order to determine the functional consequences of activated SREBP1, we further examined the gene expression levels of several key target enzymes of *SREBP1* involved in fatty acid synthesis, including *ACC1* and *FASN*. Surprisingly, no significant effects of starch level were observed in the expression of these genes. However, one-way ANOVA analysis showed that expression of *ACC1* was the highest in the HS group, which was similar to the expression of *SREBP1*. A significantly lower expression of *ACC1* and *FASN* was observed in the OE-supplemented diets, strongly indicating that OE decreased lipogenesis. In addition, OE supplementation increased the expression levels of the lipolysis gene *ATGL* and β-oxidation gene *CPT1α*. These results are consistent with a previous study showing that a Chinese olive fruit extract improved the metabolic abnormalities of mice associated with fatty liver under high fat challenge by increasing the protein expression of phosphorylated AMPK, ACC1, CPT-1, and PPARα, but downregulating the expression of mature SREBP-1c and FAS [15]. In this study, OE inhibited hepatic lipogenesis and promoted lipolysis and β-oxidation, leading to significant reductions in the content of hepatic TG and LDL-C. However, this pathway was not regulated by AMPK, and the key regulatory factors need to be further identified. Still, our study presents the first report that OE can regulate fat metabolism in aquatic animals.

At the level tested in this study (0.125%), supplementation with OE was associated with several beneficial effects. However, it is important to note that the concentration of OE supplemented into diets should be optimized, as hepatotoxic effects of some triterpenes have been reported in a dose-dependent manner. For instance, the anti-inflammatory pentacyclic triterpene oleanolic acid at about 20% caused body weight loss, inflammation, hepatocellular apoptosis, necrosis, and feathery degeneration (indicative of cholestasis) in mice [48]. Therefore, it is necessary to clarify the optimal addition level of triterpene-rich OE in the feed of aquatic animals in future studies.

## 5. Conclusions

In summary, the intake of high starch diets induced oxidative stress, inflammatory response, lipid metabolism disorder and even MLD, which significantly reduced the growth performance of largemouth bass. Supplementation with 0.125% of an olive extract obtained as a byproduct of olive oil production significantly reduced FCR, which would result in a lower farming feed cost of largemouth bass. In addition, our results demonstrate that supplementation with OE in the HS diet increased the hepatic antioxidant capacity, promoted the activation of JNK signaling pathway and decreased inflammatory responses. In this study, we present the first evidence that OE regulates hepatic lipid metabolism in fish. The protective mechanisms induced by the supplementation of OE mainly depend on inhibiting hepatic lipogenesis and promoting lipolysis and β-oxidation, leading to the prevention of hepatic TG over-accumulation in fish. A limited effect of OE was observed histologically on the repair of MLD induced by high starch in largemouth bass, but considering the short trial period and single doses tested, it would be worthwhile to further investigate the effects of different OE doses and feeding time on the growth, hepatoprotection and immune status of fish in future studies.

## Figures and Tables

**Table 1:** Formulation and composition of experimental diets (%).

Ingredients	LS	HS	LSOE	HSOE
Fish meal ^a^	30.0	30.0	30.0	30.0
Cottonseed protein concentrate ^a^	23.5	22.6	23.4	22.5
Microbial protein ^a^	4.0	4.0	4.0	4.0
Tapioca starch	5.0	5.0	5.0	5.0
Wheat flour	9.0	18.0	9.0	18.0
Wheat gluten meal	4.0	4.0	4.0	4.0
Soybean meal ^a^	2.0	-	2.0	-
Spay-dried blood cell powder	4.0	4.0	4.0	4.0
α-cellulose	4.6	-	4.6	-
Ca(H_2_PO4)_2_	1.7	1.7	1.7	1.7
Lecithin oil	2.0	2.0	2.0	2.0
Fish oil	3.5	3.5	3.5	3.5
Soybean oil	3.5	3.5	3.5	3.5
Vitamin and mineral premix ^b^	1.4	1.4	1.4	1.4
Kelp powder	1.5	0	1.5	0
*L*-Thr	0.1	0.1	0.1	0.1
*DL*-Met	0.2	0.2	0.2	0.2
Olive extract	0	0	0.125	0.125
Total	100	100	100	100
*Analyzed chemical composition (dry matter basis %)*				
Moisture	6.10	7.43	7.25	7.34
Crude protein	50.83	51.15	51.17	51.11
Crude lipid	12.36	12.33	12.44	12.41
Crude ash	10.08	10.04	9.84	9.82
Starch ^c^	9.00	14.40	9.00	14.40
Gross energy (MJ/Kg)	20.45	20.15	20.49	20.27

^a^ Fish meal: crude protein content was 68.8%; cottonseed protein concentrate: crude protein content was 61.5%; microbial protein: crude protein content was 86.7%; soybean meal: crude protein content was 44.7%. ^b^ Vitamin premix (mg/kg diet): vitamin A 20; vitamin D3 10; vitamin K3 20; vitamin E 400; vitamin B1 10; vitamin B2 15; vitamin B6 15; vitamin B12 (1%) 8; ascorbic acid (35%) 1000; calcium pantothenate 40; niacinamide 100; inositol 200; biotin (2%) 2; folic acid 10; corn gluten meal 150; choline chloride (50%) 4000. Mineral premix (mg/kg diet): CuSO_4_·5H_2_O 10; FeSO_4_·H_2_O 300; ZnSO_4_·H_2_O 200; MnSO_4_·H_2_O 100; KI (10%) 80; CoCl_2_·6H_2_O (10% Co) 5; Na_2_SeO_3_ (10% Se) 10; MgSO4·5H_2_O 2000; NaCl 100; zeolite 4995; antioxidant 200. ^c^ Starch content was estimated based on the starch content of tapioca starch (72% starch) and wheat flour (60% starch).

**Table 2:** Primer sequences for RT-qPCR.

Genes	Forward Primer (5′-3′)	Reverse Primer (5′-3′)	Tm (°C)	E-Values (%)	Accession Number
EF1α	TGCTGCTGGTGTTGGTGAGTT	TTCTGGCTGTAAGGGGGCTC	60.4	102.8	119901934
ACC1	ATCCCTCTTTGCCACTGTTG	GAGGTGATGTTGCTCGCATA	57.5	102.2	119896388
FASN	TGTGGTGCTGAACTCTCTGG	CATGCCTAGTGGGGAGTTGT	57.5	102.1	119915567
ATGL	CCATGATGCTCCCCTACACT	GGCAGATACACTTCGGGAAA	58	99.1	119893301
CPT1α	CATGGAAAGCCAGCCTTTAG	GAGCACCAGACACGCTAACA	60.0	98.8	119893292
TNFα	CTTCGTCTACAGCCAGGCATCG	TTTGGCACACCGACCTCACC	63	105.7	119906688
IL1β	CGTGACTGACAGCAAAAAGAG	GATGCCCAGAGCCACAGTTC	59.4	101.3	119914255
TGFβ1	GCTCAAAGAGAGCGAGGATG	TCCTCTACCATTCGCAATCC	59	95.6	119882881
IL10	CGGCACAGAAATCCCAGAGC	CAGCAGGCTCACAAAATAAACA	62.1	113.6	119885912
SREBP1	AGTCTGAGCTACAGCGACAAGG	TCATCACCAACAGGAGGTCACA	61	98.1	119888831

EF1α, elongation factor-1α; ACC1, acetyl-CoA carboxylase 1; FASN, fatty acid synthase; ATGL, adipose triglyceride lipase; CPT1α, carnitine palmitoyltransferase 1α; TNFα, tumor necrosis factor α; TGFβ1, transforming growth factor β1. SREBP1, Sterol-regulatory element binding protein 1.

**Table 3:** Effects of experimental diets on the growth performance in largemouth bass.

	OE (%)	Level of Dietary Starch (%)		OE Level	*P* Values		
		9.0	14.4		OE Level	Starch Level	Interaction
IBW ^1^	-	35.98 ± 0.21		-	-	-	-
FBW ^2^	0	99.98 ± 1.68	97.66 ± 1.69	98.82 ± 1.18	0.223	0.002	0.008
	0.125	109.59 ± 1.77	93.34 ± 2.68	101.46 ± 3.91			
	starch level	104.78 ± 2.41 ^B^	95.50 ± 1.72 ^A^				
SR ^3^	0	96.67 ± 1.67	96.67 ± 1.67	96.67 ± 1.05	1.000	1.000	1.000
	0.125	96.67 ± 1.67	96.67 ± 1.67	96.67 ± 1.05			
	starch level	96.67 ± 1.05	96.67 ± 1.05				
SGR ^4^	0	1.94 ± 0.03	1.91 ± 0.03	1.93 ± 0.02	0.272	0.002	0.008
	0.125	2.12 ± 0.03	1.82 ± 0.06	1.97 ± 0.07			
	starch level	2.03 ± 0.44 ^B^	1.86 ± 0.04 ^A^				
FCR ^5^	0	1.01 ± 0.01	0.96 ± 0.01	0.98 ± 0.01 ^b^	0.002	0.005	0.327
	0.125	0.95 ± 0.01	0.93 ± 0.01	0.94 ± 0.01 ^a^			
	starch level	0.98 ± 0.01^B^	0.94 ± 0.01 ^A^				
FR ^6^	0	0.44 ± 0.01	0.42 ± 0.01	0.43 ± 0.01	0.108	0.000	0.010
	0.125	0.46 ± 0.01	0.39 ± 0.01	0.42 ± 0.02			
	starch level	0.45 ± 0.00 ^B^	0.40 ± 0.01 ^A^				

Two-way ANOVA was used to analyze the significant differences among treatment means based on starch level (9.0 and 14.4%) and OE level (0 and 0.125%). Different superscript lowercase letters “a” or “b” denote significant differences (*p* < 0.05) among experimental groups fed different OE levels; different capital letters “A” or “B” denote significant differences (*p* < 0.05) between groups with different starch levels. ^1^ IBW: initial body weight. ^2^ FBW: final body weight. ^3^ SR (survival rate, %) = 100 × final fish number/initial fish number. ^4^ SGR (specific growth rate, %/d) = 100 × [Ln (FBW)−Ln (IBW)]/days. ^5^ FCR (feed conversion ratio) = feed intake/(W_f_ + W_d_ − W_i_). W_f_ is the final total weight, W_d_ is the total weight of dead fish, and W_i_ is the initial total weight. The same below. ^6^ FR (feeding rate, %) = 100 × feed intake/[(W_f_ + W_i_ + W_d_)/2]/days.

**Table 4:** Effects of experimental diets on morphometric parameters in largemouth bass.

	OE (%)	Level of Dietary Starch (%)		OE Level	*P* Values		
		9.0	14.4		OE Level	Starch Level	Interaction
CF ^1^	0	1.96 ± 0.10	1.75 ± 0.03	1.85 ± 0.06 ^b^	0.004	0.066	0.054
	0.125	1.68 ± 0.02	1.68 ± 0.03	1.68 ± 0.02 ^a^			
	starch level	1.82 ± 0.06	1.72 ± 0.02				
VSI ^2^	0	7.14 ± 0.10	7.60 ± 0.23	7.37 ± 0.13	0.142	0.001	0.110
	0.125	7.01 ± 0.22	8.30 ± 0.29	7.66 ± 0.22			
	starch level	7.07 ± 0.12 ^A^	7.95 ± 0.20 ^B^				
HIS ^3^	0	1.72 ± 0.15	2.54 ± 0.14	2.13 ± 0.13 ^a^	0.007	0.000	0.842
	0.125	2.05 ± 0.12	3.04 ± 0.22	2.57 ± 0.17 ^b^			
	starch level	1.88 ± 0.10 ^A^	2.79 ± 0.14 ^B^				
VAI ^4^	0	1.46 ± 0.07	1.74 ± 0.20	1.60 ± 0.11	0.115	0.348	0.489
	0.125	1.85 ± 0.17	1.90 ± 0.21	1.88 ± 0.13			
	starch level	1.66 ± 0.10	1.82 ± 0.14				

Two-way ANOVA was used to analyze the significant differences among treatment means based on starch level (9.0 and 14.4%) and OE level (0 and 0.125%). Different superscript lowercase letters “a” or “b” denote significant differences (*p* < 0.05) among experimental groups fed different OE levels; different capital letters “A” or “B” denote significant differences (*p* < 0.05) between groups with different starch levels. ^1^ CF (condition factor) = 100 × (body weight, g)/(body length, cm)^3^. ^2^ VSI (viscerosomatic index, %) = 100 × visceral weight/whole body weight. ^3^ HSI (hepatosomatic index, %) = 100 × liver weight/whole body weight. ^4^ VAI (visceral adipose index, %) = 100 × visceral adipose weight/whole body weight.

**Table 5:** Effects of experimental diets on the composition of the whole body in largemouth bass.

	OE (%)	Level of Dietary Starch (%)		OE Level	*P* Values		
		9.0	14.4		OE Level	Starch Level	Interaction
Moisture	0	73.39 ± 1.14	70.60 ± 0.18	72.00 ± 0.81 ^b^	0.004	0.066	0.054
	0.125	69.87 ± 0.49	69.87 ± 0.49	69.74 ± 0.26 ^a^			
	starch level	71.63 ± 0.96	70.11 ± 0.27				
Crude protein	0	16.58 ± 0.15	16.94 ± 0.06	16.76 ± 0.11	0.142	0.001	0.110
	0.125	16.70 ± 0.15	16.13 ± 0.05	16.42 ± 0.15			
	starch level	16.64 ± 0.10	16.54 ± 0.19				
Crude lipid	0	5.37 ± 0.84	7.93 ± 0.22	6.65 ± 0.69 ^a^	0.007	0.000	0.842
	0.125	9.24 ± 0.36	9.16 ± 0.23	9.20 ± 0.19 ^b^			
	starch level	7.31 ± 0.96	8.54 ± 0.31				

Two-way ANOVA was used to analyze the significant differences among treatment means based on starch level (9.0 and 14.4%) and OE level (0 and 0.125%). Different superscript lowercase letters “a” or “b” denote significant differences (*p* < 0.05) among experimental groups fed different OE levels.

**Table 6:** Effects of experimental diets on hematological parameters in largemouth bass.

	OE(%)	Level of Dietary Starch (%)		OE Level	*P* Values		
		9.0	14.4		OE Level	Starch Level	Interaction
Glucose (mM/L)	0	4.82 ± 0.40	4.98 ± 0.70	4.90 ± 0.30	0.097	0.053	0.043
	0.125	5.05 ± 0.37	3.19 ± 0.31	4.12 ± 0.33			
	Starch level	4.93 ± 0.26	4.08 ± 0.36				
TG (mM/L)	0	4.31 ± 0.43	6.59 ± 0.98	5.45 ± 0.60	0.895	0.598	0.038
	0.125	5.82 ± 0.77	4.68 ± 0.54	5.25 ± 0.48			
	starch level	5.07 ± 0.47	5.64 ± 0.59				
TC (mM/L)	0	6.34 ± 0.29	7.13 ± 0.48	6.74 ± 0.29^a^	0.000	0.587	0.167
	0.125	8.73 ± 0.57	8.33 ± 0.40	8.53 ± 0.34^b^			
	starch level	7.53 ± 0.44	7.73 ± 0.34				
HDL-C (mM/L)	0	1.47 ± 0.23	1.20 ± 0.21	1.33 ± 0.15^a^	0.013	0.742	0.511
	0.125	1.86 ± 0.29	2.05 ± 0.25	1.95 ± 0.18^b^			
	starch level	1.66 ± 0.18	1.59 ± 0.19				
HDL-C/TC	0	0.23 ± 0.03	0.17 ± 0.03	0.20 ± 0.02	0.363	0.522	0.190
	0.125	0.21 ± 0.03	0.22 ± 0.03	0.21 ± 0.02			
	starch level	0.22 ± 0.02	0.19 ± 0.02				
LDL-C (mM/L)	0	1.77 ± 0.22	2.56 ± 0.21	2.16 ± 0.18	0.591	0.059	0.100
	0.125	2.22 ± 0.20	2.26 ± 0.18	2.24 ± 0.13			
	starch level	2.00 ± 0.16	2.41 ± 0.14				
LDL-C/TC	0	0.28 ± 0.03	0.37 ± 0.03	0.32 ± 0.03^b^	0.021	0.154	0.109
	0.125	0.26 ± 0.02	0.25 ± 0.02	0.25 ± 0.01^a^			
	starch level	0.27 ± 0.02	0.31 ± 0.02				

Two-way ANOVA was used to analyze the significant differences among treatment means based on starch level (9.0 and 14.4%) and OE level (0 and 0.125%). Different superscript lowercase letters “a” or “b” denote significant differences (*p* < 0.05) among experimental groups fed different OE levels.

**Table 7:** Effects of experimental diets on hematological liver function in largemouth bass.

	OE (%)	Level of Dietary Starch (%)		OE Level	*P* Values		
		9.0	14.4		OE Level	Starch Level	Interaction
AKP (U/L)	0	41.15 ± 3.51	92.49 ± 8.46	66.82 ± 7.97	0.551	0.000	0.001
	0.125	69.61 ± 9.19	70.34 ± 5.91	69.98 ± 9.86			
	starch level	55.38 ± 6.01 ^A^	81.42 ± 5.75 ^B^				
AST (U/L)	0	4.96 ± 0.58	14.58 ± 2.38	9.86 ± 2.30	0.317	0.000	0.087
	0.125	6.22 ± 0.85	10.37 ± 1.11	8.87 ± 1.17			
	starch level	5.33 ± 1.13 ^A^	12.89 ± 1.77 ^B^				
ALT (U/L)	0	4.55 ± 0.51	15.86 ± 1.65	9.99 ± 1.85 ^b^	0.022	0.000	0.000
	0.125	6.98 ± 0.91	8.50 ± 0.74	8.14 ± 1.00 ^a^			
	starch level	5.73 ± 1.02 ^A^	12.25 ± 1.45 ^B^				

Two-way ANOVA was used to analyze the significant differences among treatment means based on starch level (9.0 and 14.4%) and OE level (0 and 0.125%). Different superscript lowercase letters “a” or “b” denote significant differences (*p* < 0.05) among experimental groups fed different OE levels; different capital letters “A” or “B” denote significant differences (*p* < 0.05) between groups with different starch levels.

**Table 8:** Effects of experimental diets on hepatic antioxidant parameters in largemouth bass.

	OE (%)	Level of Dietary Starch (%)		OE Level	*P* Values		
		9.0	14.4		OE Level	Starch Level	Interaction
ROS (U/mg prot)	0	64.42 ± 4.61	86.41 ± 4.42	75.41 ± 4.20	0.105	0.000	0.101
	0.125	64.33 ± 3.73	102.84 ± 6.37	83.59 ± 6.12			
	starch level	64.37 ± 2.86 ^A^	94.62 ± 4.30 ^B^				
T-AOC (µM/g prot)	0	69.12 ± 5.80	95.70 ± 8.69	82.41 ± 6.10	0.749	0.185	0.009
	0.125	91.01 ± 7.21	81.60 ± 6.37	86.31 ± 4.80			
	starch level	80.07 ± 5.29	88.65 ± 5.51				
CAT (U/mg prot)	0	49.61 ± 3.02	47.81 ± 4.39	48.71 ± 2.58 ^b^	0.000	0.000	0.000
	0.125	49.83 ± 3.70	8.17 ± 1.48	29.00 ± 5.71 ^a^			
	starch level	49.72 ± 2.31 ^B^	27.99 ± 5.59^A^				
GSH-Px (U/ug prot)	0	4.14 ± 0.70	4.57 ± 0.74	4.35 ± 0.49	0.137	0.815	0.642
	0.125	4.87 ± 0.54	5.24 ± 0.42	5.06 ± 0.33			
	starch level	4.50 ± 0.44	4.90 ± 0.42				
SOD (U/mg prot)	0	195.90 ± 11.13	192.83 ± 11.57	194.37 ± 7.76 ^b^	0.004	0.038	0.104
	0.125	185.02 ± 9.71	144.04 ± 7.72	164.53 ± 7.99 ^a^			
	starch level	190.46 ± 7.27 ^B^	168.44 ± 9.21^A^				
MDA (nM/mg prot)	0	4.48 ± 0.49	2.70 ± 0.85	3.53 ± 0.54 ^b^			
	0.125	1.22 ± 0.16	2.17 ± 0.45	1.70 ± 0.26 ^a^	0.002	0.452	0.020
	starch level	2.75 ± 0.49	2.43 ± 0.47				
SOD/MDA	0	47.5 ± 6.08	103.8 ± 21.79	77.52 ± 13.74^a^	0.004	0.819	0.002
	0.125	166.93 ± 19.51	98 ± 23.14	132.47 ± 17.12^b^			
	starch level	111.2 ± 19.00	100.90 ± 15.37				

Two-way ANOVA was used to analyze the significant differences among treatment means based on starch level (9.0 and 14.4%) and OE level (0 and 0.125%). Different superscript lowercase letters “a” or “b” denote significant differences (*p* < 0.05) among experimental groups fed different OE levels; different capital letters “A” or “B” denote significant differences (*p* < 0.05) between groups with different starch levels.

**Table 9:** Effects of experimental diets on hepatic lipid metabolism parameters in largemouth bass.

	OE (%)	Level of Dietary Starch (%)		OE Level	*P* Values		
		9.0	14.4		OE Level	Starch Level	Interaction
TG (mM/g prot)	0	0.39 ± 0.06	0.32 ± 0.05	0.36 ± 0.05 ^b^	0.010	0.838	0.197
	0.125	0.20 ± 0.03	0.25 ± 0.05	0.22 ± 0.03 ^a^			
	starch level	0.30 ± 0.04	0.29 ± 0.05				
TC (mM/g prot)	0	0.16 ± 0.02	0.15 ± 0.01	0.16 ± 0.01	0.426	0.286	0.041
	0.125	0.12 ± 0.01	0.17 ± 0.01	0.15 ± 0.01			
	starch level	0.14 ± 0.01	0.16 ± 0.01				
LDL-C (µM/g prot)	0	38.03 ± 3.01	42.67 ± 3.04	40.35 ± 2.15 ^b^	0.010	0.056	0.426
	0.125	26.06 ± 2.29	35.20 ± 3.30	30.63 ± 2.27 ^a^			
	starch level	32.04 ± 2.39	38.94 ± 2.37				
LDL-C/TC	0	0.24 ± 0.02	0.21 ± 0.02	0.26 ± 0.17	0.050	0.504	0.273
	0.125	0.22 ± 0.02	0.21 ± 0.22	0.22 ± 0.13			
	starch level	0.23 ± 0.02	0.25 ± 0.02				
TBA (µM/g prot)	0	3.14 ± 0.21	3.47 ± 0.91	3.29 ± 0.42 ^b^	0.001	0.917	0.551
	0.125	1.66 ± 0.14	1.43 ± 0.14	1.54 ± 0.10 ^a^			
	starch level	2.45 ± 0.24	2.45 ± 0.52				
Liver lipid (%)	0	1.99 ± 0.01	2.01 ± 0.16	2.03 ± 0.06	0.606	0.007	0.006
	0.125	1.66 ± 0.10	2.27 ± 0.04	1.95 ± 0.15			
	starch level	1.77 ± 0.10^A^	2.16 ± 0.07^B^				

Two-way ANOVA was used to analyze the significant differences among treatment means based on starch level (9.0 and 14.4%) and OE level (0 and 0.125%). Different superscript lowercase letters “a” or “b” denote significant differences (*p* < 0.05) among experimental groups fed different OE levels; different capital letters “A” or “B” denote significant differences (*p* < 0.05) between groups with different starch levels.

## Data Availability

The data presented in this study are available on request from the corresponding author. The data are not publicly available due to containing information that could compromise the privacy of research participants.
